# The Important Role of Social Media During the COVID-19 Epidemic

**DOI:** 10.1017/dmp.2020.330

**Published:** 2020-09-10

**Authors:** Qilin Tang, Kai Zhang, Yan Li

**Affiliations:** School of Basic Medical Sciences, Hebei University of Chinese Medicine, Hebei, Shijiazhuang, China; Department of Acupuncture and Moxibustion, Tianjin Gong An Hospital, Tianjin, China

**Keywords:** coronavirus, COVID-19, health personnel, social media

The 2019 coronavirus disease (COVID-19) epidemic has received close attention from governments, researchers, and the public in various countries.^1,2^ In this case, billions of people are eager to get information about COVID-19 through social media. The rapid dissemination of topics and information related to COVID-19 has affected the behavior of the public during the epidemic. Today, more than 2.9 billion people use social media regularly.^3^ These social media have an amazing spread speed, coverage, and penetration rate. During the COVID-19 epidemic, the social media platforms play an important role in dissemination information.^3^

## PROMOTE RELIABLE INFORMATION AND COMBAT MISINFORMATION

The World Health Organization (WHO) has found that the outbreak of COVID-19 and the response measures are accompanied by abundant information, and it is difficult to find reliable sources and reliable guidance. For rumors and false information spread on social media, it is necessary to coordinate the search for sources, identify, and reduce their spread.^4^ A study evaluating the number of times people watch COVID-19 medical videos on YouTube found that independent users were more likely to post misleading videos than useful ones (60.0% vs 21.5%, *P* = 0.009).^5^ The actions of government agencies and social media giants have shown that public-private cooperation to identify, fact-check, and even delete false or outdated information may be an effective way to prevent these online information from hindering or even worsening public health efforts.^6^ Social media operators can monitor high-traffic information and combine artificial intelligence to remove misleading information in a timely manner.

As clinicians in China, we often hear patients say that they have wanted to see a doctor for a long time. When they saw some reports in the media, they did not dare to go out or even come to the hospital. Some patients have said: “COVID-19 is a terrible infectious disease, most patients will die after infection,” or “the virus is still in the air, I dare not open the window.” Although the Health Committee of the People’s Republic of China recommends that people do not need to wear masks when there is no crowd outdoors,^7^ some people are afraid to take off the masks because they are worried about being infected with airborne viruses. In fact, social media play a vital role in the dissemination of public health knowledge. However, during the epidemic, it is sometimes abused to spread unrealistic news, which may cause mental health problems.^8^ Therefore, social media need to publish and update information about the epidemic in a timely manner, and popularize knowledge through the government and medical professionals to help guide the public correctly and stabilize public sentiment.

## PROMOTE THE HEALTHY DEVELOPMENT OF SOCIAL MEDIA

The WHO, academic institutions, and other official health institutions should consider using influential social media to disseminate accurate medical information to the general public. The information quality of social media should also be monitored. Ideally, established health care experts should ensure that potential misinformation is not disseminated. For global institutions, such as the WHO, the dissemination of correct information in different languages could be considered, especially in developing countries.
